# Higher serum uric acid to HDL-cholesterol ratio is associated with onset of non-alcoholic fatty liver disease in a non-obese Chinese population with normal blood lipid levels

**DOI:** 10.1186/s12876-022-02263-4

**Published:** 2022-04-21

**Authors:** Wentao Zhu, An Liang, Pei Shi, Songsong Yuan, Ying Zhu, Jiwei Fu, Ting Zheng, Zhilong Wen, Xiaoping Wu

**Affiliations:** Department of Infectious Diseases, The First Affiliated Hospital of Nanchang University, No. 17 Yongwai Street, Donghu District, Nanchang, China

**Keywords:** Serum uric acid to HDL-cholesterol ratio, Non-alcoholic fatty liver disease, Non-obese, Longitudinal study

## Abstract

**Background:**

Recent studies have demonstrated the presence of associations between metabolic syndrome and the onset of nonalcoholic fatty liver disease (NAFLD). Metabolic syndrome, in turn, has been found to be linked to high serum uric acid to HDL-cholesterol ratios (UHR). However, the relationship between UHR values and the occurrence of NAFLD in non-obese individuals remains unknown. The present study aimed to examine the possible correlation between UHR values and NAFLD onset among a non-obese Chinese population without dyslipidemia, as well as comparing the predictive value of UHR versus other NAFLD onset predictors.

**Methods:**

A total of 9837 non-obese patients, with normal blood lipid levels, were included in a 5-year retrospective cohort study, and the onset of NAFLD in these patients was diagnosed by liver ultrasound.

**Results:**

Out of the 9837 patients, 855 were diagnosed with NAFLD during the 5-year follow-up period, for an overall total prevalence of 8.7% at the end of the study period. Across quintiles 1, 2, 3, 4 and 5 of UHR (respectively, ratios of ≤ 120.88, 120.89–154.01, 154.02–189.91, 189.92–240.46, and ≥ 240.47), the prevalence of NAFLD among the patients increased from 2.4%, 5%, 7.9%, 10.3%, and 17.8%, respectively. After adjustments for age, gender, liver and kidney functional markers, as well as metabolic indicators, multivariate Cox proportional hazard regression analysis demonstrated that the hazard ratio (HR) was the highest in quintile 5, at 1.76 (1.12–2.75), and the lowest in quintile 1. The area under the curve (AUC) for UHR (0.690) was higher than that for serum uric acid (UA, 0.666) and HDL-C (0.636), suggesting the predictive ability of UHR for NAFLD onset was better than either alone. This finding was further supported by the presence of an independent association between UHR and NAFLD, even within the normal range of UA and HDL-C; the HR (95% confidence interval, CI) for NAFLD was 1.002 (1.000–1.004). Compared with other significant predictors, AUC for UHR (0.67) was similar to that of low-density lipoprotein cholesterol (LDL-C)/high-density lipoprotein cholesterol (HDL-C, 0.68), non-high-density lipoprotein cholesterol (NHDL-C)/HDL-C (0.68) and alanine aminotransferase (ALT)/aspartate aminotransferase (AST) ratios (0.7), and was higher than that of LDL-C (0.63), remnant cholesterol (RC,0.59), and albumin (ALB)/alkaline phosphatase (ALP) ratio (0.61). The sensitivity of UHR (71%) was the highest among all indicators. In the subgroup with ALT < 40U/L, the AUC for UHR was 0.70, which was the highest among all predictors; among ALT > 40U/L, UHR was able to predict the occurrence of NAFLD (AUC = 0.61, p = 0.007), which was not the case for RC (P = 0.441), ALB/ALP (P = 0.419), and ALT/AST (P = 0.159).

**Conclusions:**

UHR serve as an inexpensive and reliable predictor of NAFLD onset in non-obese Chinese people with normal blood lipid levels, allowing for identification of individuals at high risk for NAFLD.

## Background

Non-alcoholic fatty liver disease (NAFLD) is a common chronic liver disease, characterized by lipid deposition in hepatocytes. Its clinical manifestations include hepatic steatosis, steatohepatitis, fibrosis and cirrhosis, and it is significantly associated with the occurrence of hepatocellular carcinoma [1–3]. Obesity has long been considered as a major risk factor for NAFLD [4]; however, the disease has also been commonly observed in non-obese individuals [5–7]. In a large Chinese retrospective study, 14.4% out of 16,173 non-obese patients, all of whom did not initially have NAFLD, were eventually diagnosed with NAFLD after 5 years of follow-up [8]. Additionally, other studies have shown that the prevalence of steatohepatitis and fibrosis in non-obese NAFLD patients is similar to that in obese NAFLD patients [9], and that non-obese NAFLD patients are also at high risk of Type 2 diabetes and cardiovascular disease [10].

No specific symptoms are present in most patients in the early stages of NAFLD. As a result, developing methods to detect NAFLD in non-obese patients has become crucial. Currently, liver biopsy is the gold standard for diagnosing NAFLD, but it is associated with significant limitations, due to the invasive nature of the operation, along with the risk of sampling errors [11]. Therefore, multiplestudies have attempted to find a simpler, less invasive detection indicatorby investigating various serum markers. One such indicator is serum uric acid (UA), a metabolite of purines in the liver. It has been considered as a possible predictor for liver damage severity in NAFLD [10], in which studies have shown a significant positive correlation between UA level and the risk of developing NAFLD [12]. Furthermore, Kosekli et al. have proposed that the UA to high density lipoprotein cholesterol (HDL-C) ratio (UHR) could be used as a useful indicator for diagnosing hepatic steatosis [13]. However, limited information is currently available with respect to the relationship between UHR and NAFLD onset in non-obese people, with normal lipid levels. Therefore, our study aims to determine whether UHR can be used to independently assess NAFLD risk in non-obese Chinese individuals, with normal lipid levels. We found that higher UHR ratios were independently associated with NAFLD onset, and that its predictive value was equally as strong, or stronger, than for other commonly-used ratios, such as low density lipoprotein cholesterol (LDL-C)/HDL-C, etc.

## Method

### Study design and population

This study was a secondary analysis of a longitudinal cohort study, which enrolled 16,173 non-obese Chinese individuals in Wenzhou People’s Hospital, from January 2010-December 2014. The data were from the Dryad database and shared by Sun et al. [8]. Patients fulfilling the following inclusion criteria at baseline were included: (i) No excessive drinking (female: < 70 g/week, male: < 140 g/week), (ii) No viral hepatitis, autoimmune hepatitis or other known causes of chronic liver disease were present, (iii) No antihypertensive, antidiabetic, or lipid-lowering agents were taken at baseline, (iv) Body mass index (BMI) < 25 kg/m^2^ (non-obese), (v) LDL-C within normal reference range (≤ 3.12 mmol/L), and (vi) complete follow-up data. Exclusion criteria involved patients with dyslipidemia (total cholesterol (TC) > 5.2 mmol/L, triglyceride (TG) > 1.7 mmol/L, LDL-C > 3.12 mmol/L, HDL-C < 1.03 mmol/L). Upon application of both inclusion and exclusion criteria, 9837 subjects were included in this study. Ethical approval was already provided for the initial longitudinal cohort study; therefore, it was not necessary to obtain ethical approval for the current study.

### Data collection

Height, body weight, gender, age, as well as systolic (SBP) and diastolic blood pressure (DBP) were recorded using a standardized self-filled spreadsheet. Blood pressure was measured with automatic sphygmomanometer, in which the subjects were sitting within a quiet environment. BMI was calculated as the weight (kg), divided by the square of their height (m^2^). Venous blood was collected after overnight fasting, and analyzed with the Abbott AxSYM automatic biochemical analyzer by trained medical personnel, measuring the following laboratory parameters: TC, TG, LDL-C, HDL-C, UA, blood urea nitrogen (BUN), creatinine (Cr), alanine aminotransferase (ALT), aspartate aminotransferase (AST), albumin (ALB), fasting plasma glucose (FPG), total bilirubin (TB), globulin (GLB; consists of a variety of glycoproteins, along with serum proteins, including immunoglobulin and complement), total protein (TP; consists of GLB + ALB).

### Diagnosis of NAFLD and follow-up

During the 5-year follow-up period, subjects were evaluated annually by liver ultrasound for NAFLD, and NAFLD diagnosis was based on criteria suggested by the Chinese Liver Disease Association in 2010 [14], entailing diffuse hyper-echogenicity of the liver compared to spleen and kidney, combined with any of the following: (i) Unclear display of intrahepatic structure, (ii) Enlarged liver with a round and blunt border, (iii) Right liver lobe and diaphragm beingunclear or incomplete, or (iv) Weakened hepatic blood flow signal, but in association with normal blood flow distribution. UHR was calculated as serum UA levelsdivided by HDL-C levels (both in mmol/L).

### Statistical analysis

To assess the association between UHR and prevalence of NAFLD, all subjectswere divided into 5 quintile groups, as follows: Q1with UHR ≤ 120.88, Q2: 120.89–154.01, Q3: 154.02–189.91, Q4: 189.92–240.46, and Q5 ≥ 240.47. Continuous variables were displayed as mean ± standard deviation (SD), and categorical variables as medians (quartile). Classification data was presented as frequency and proportion. Significant differences between groups were evaluated by a non-parametric test and one-way analysis of variance (ANOVA) for continuous variables, and χ^2^ test for categorical variables. P < 0.05 was considered statistically significant. Kaplan–Meier analysis was used to calculate the cumulative hazard of NAFLD over time, and hazard ratios (HRs) and 95% confidence intervals (CI) were determined based on the Cox proportional hazards regression model. Four models were used in this study, in which model 1 was the original model without adjustment, 2 was adjusted only for age and gender, 3 adjusted for the same parameters as model 2, plus major liver and kidney functional markers (alkaline phosphatase [ALP], ALT, AST, ALB, TP, TB, BUN and Cr), and 4 adjusted for the same parameters as model 3, plus metabolic indicators (FPG, TC, TG, LDL, BMI and SBP). UA and HDL-C were excluded from the models, in order to avoid possible confounding effects, as both parameters were included in UHR. The best model out of the 4 was determined using receiver operator characteristic (ROC) curve analyses, and sensitivity analysis tests were performed to exclude any possible confounding interactions between UA and HDL-C levels on UHR. In order to assess the ability of UHR to detect NAFLD, UHR was compared to other significant recently-proposed predictors found in the literature, such as LDL-C/HDL-C, ALB/ALP, ALT/AST, and non-HDL cholesterol (NHDL-C)/HDL-C ratios, as well as remnant cholesterol (RC), through ROC curve analyses. Additionally, some subgroup stratified analyses (gender and ALT) were conducted, in order to account for the possibility of different conditions affecting the correlation between the aforementioned indicators and NAFLD onset. All data were evaluated using SPSS 22.0 (SPSS Inc., Chicago, IL, USA).

## Results

### Characteristics of study subjects

The mean age of the study subjects was 42.5 ± 14.7 years, and an almost equal proportion of male and female patients were present (50.9% vs. 49.1%). The overall prevalence rate for NAFLD at the end of the 5-year follow-up period was 8.7% out of the 9837 patients in the study. The subjects were divided into 5 quintile groups, according to their UHR values, and the basic characteristics for the patients within each of those groups were displayed in Table 1. There, subjects with higher UHR values were older, taller, and heavier, compared to those with lower UHR. Furthermore, those subjects also had higher values for the biochemical and circulatory parameters listed in Table 1, except for GLB, TC, and HDL-C, where the values either decreased, or stayed at the same level.

**Table 1:** Baseline characteristics of the study participants (N = 9837)

Characteristics	Quartiles of UHR					*P* value
	Q1 (≤ 120.88)	Q2 (120.89–154.01)	Q3 (154.02–189.91)	Q4 (189.92–240.46)	Q5 (≥ 240.47)	
Gender Male/Female (n)	902/1065	924/1045	957/1009	1086/882	1136/831	** < 0.001**
Age (years) Mean ± SD	41.83 ± 12.27	41.99 ± 14.58	42.29 ± 14.66	43.16 ± 15.09	43 ± 14.87	**0.011**
NAFLD onset n (%)	47 (2.4%)	98 (5%)	156 (7.9%)	203 (10.3%)	351 (17.8%)	** < 0.001**
ALP (U/L) Median (Quartile)	58.00 (49.00–69.00)	62.00 (52.00–76.00)	68.00 (56.00–82.00)	71.00 (60.00–85.00)	74.00 (62.00–87.00)	** < 0.001**
ALT (U/L) Median (Quartile)	13.00 (11.00–17.00)	14.00 (11.00–19.00)	15.00 (12.00–21.00)	17.00 (12.00–22.00)	18.00 (13.00–24.00)	** < 0.001**
AST (U/L) Median (Quartile)	19.00 (17.00–22.00)	20.00 (17.00–23.00)	21.00 (18.00–24.00)	21.00 (18.00–25.00)	22.00 (19.00–26.00)	** < 0.001**
TP (g/L) Mean ± SD	73.28 ± 4.07	73.58 ± 3.99	73.83 ± 4.04	74.04 ± 4.12	74.18 ± 4.22	** < 0.001**
ALB (g/L) Mean ± SD	43.87 ± 2.72	44.09 ± 2.60	44.38 ± 2.83	44.61 ± 2.73	44.73 ± 2.80	** < 0.001**
GLB (g/L) Mean ± SD	29.44 ± 3.69	29.57 ± 3.70	29.53 ± 3.93	29.47 ± 3.82	29.49 ± 4.18	** < 0.001**
TB (μmol/L) Median (Quartile)	11.00 (8.00–14.00)	11.00 (9.00–14.00)	11.00 (9.00–14.00)	12.00 (9.00–16.00)	12.00 (10.00–16.00)	** < 0.001**
BUN (mmol/L) Median (Quartile)	4.20 (3.50–5.05)	4.20 (3.50–5.10)	4.30 (3.51–5.10)	4.40 (3.60–5.26)	4.40 (3.70–5.30)	** < 0.001**
Cr (μmol/L) Median (Quartile)	66.00 (59.00–72.00)	68.00 (60.50–76.00)	72.00 (62.00–86.00)	83.00 (68.00–95.00)	90.00 (79.00–101.00)	** < 0.001**
UA (mmol/L) Mean ± SD	176.34 ± 34.98	221.96 ± 35.03	254.95 ± 42.10	296.73 ± 46.36	369.55 ± 61.84	** < 0.001**
FPG (mmol/L) Mean ± SD	4.97 ± 0.58	5.02 ± 0.70	5.08 ± 0.82	5.12 ± 0.73	5.17 ± 0.64	** < 0.001**
TC (mmol/L) Mean ± SD	4.39 ± 0.51	4.35 ± 0.51	4.36 ± 0.54	4.33 ± 0.54	4.27 ± 0.54	** < 0.001**
TG (mmol/L) Mean ± SD	0.81 ± 0.25	0.89 ± 0.28	0.96 ± 0.30	1.05 ± 0.31	1.13 ± 0.30	** < 0.001**
HDL-C (mmol/L) Median (Quartile)	1.79 (1.62–1.99)	1.60 (1.45–1.78)	1.41 (1.31–1.65)	1.37 (1.24–1.52)	1.23 (1.13–1.36)	** < 0.001**
LDL-C (mmol/L) Median (Quartile)	1.99 (1.71–2.27)	2.07 (1.81–2.35)	2.17 (1.88–2.43)	2.19 (1.90–2.46)	2.25 (1.94–2.52)	** < 0.001**
Height (m) Mean ± SD	160.53 ± 5.49	161.56 ± 6.58	163.73 ± 7.44	166.66 ± 7.69	169.21 ± 7.26	** < 0.001**
Weight (kg) Mean ± SD	52.04 ± 5.85	53.76 ± 6.81	56.36 ± 7.77	59.33 ± 7.93	63.01 ± 7.99	** < 0.001**
BMI (kg/m^2^) Median (Quartile)	20.10 (18.87–21.52)	20.53 (19.27–21.94)	21.04 (19.56–22.46)	21.41 (19.86–22.88)	22.21 (20.66–23.59)	** < 0.001**
SBP (mmHg) Median (Quartile)	112.00 (103.00–120.00)	113.00 (104.00–122.00)	116.00 (106.00–127.00)	119.00 (109.00–131.00)	122.00 (113.00–134.00)	** < 0.001**
DBP (mmHg) Median (Quartile)	68.00 (62.00–74.00)	68.00 (62.00–75.00)	70.00 (64.00–78.00)	72.00 (65.00–80.00)	73.00 (67.00–85.25)	** < 0.001**

### Establishing the association between UHR and NAFLD onset

**Fig. 1:**
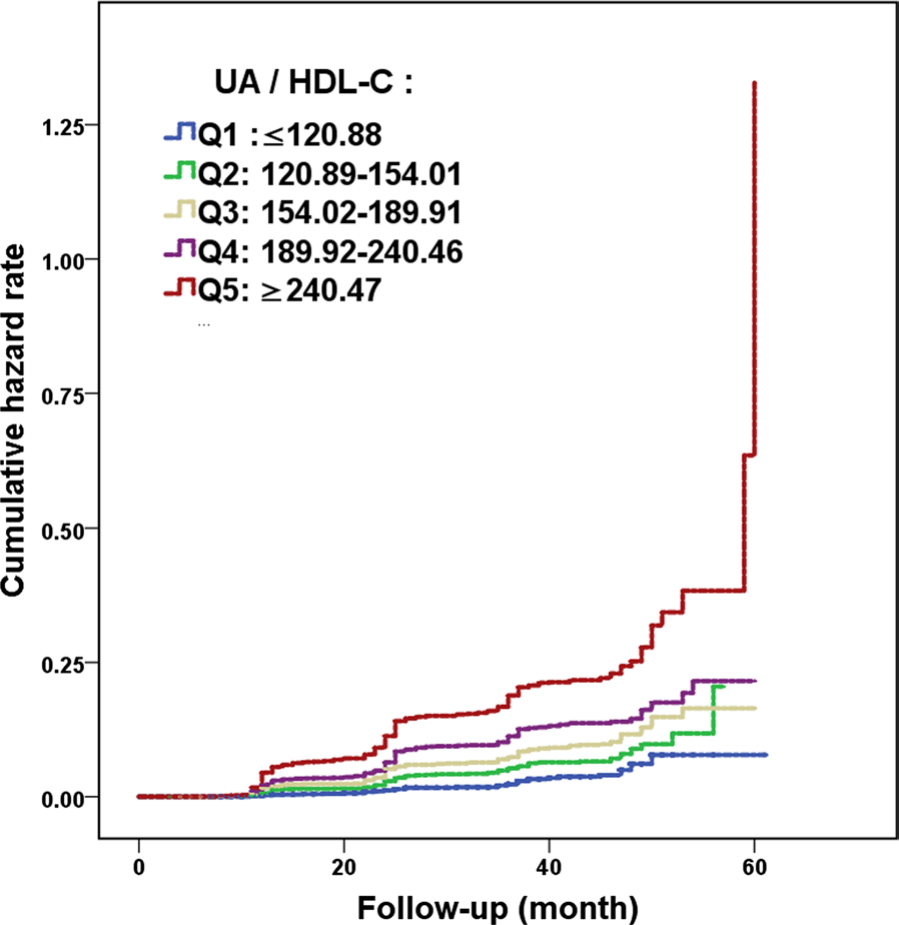
Kaplan–Meier curves for the 5 quintiles of serum uric acid (UA) to HDL-cholesterol (HDL-C) ratios (UHR) (Q1-5). A positive correlation is present with respect to increasing UHR values and increasing cumulative incidence of NAFLD among non-obese individuals with normal blood lipid levels. P < 0.001

**Fig. 2:**
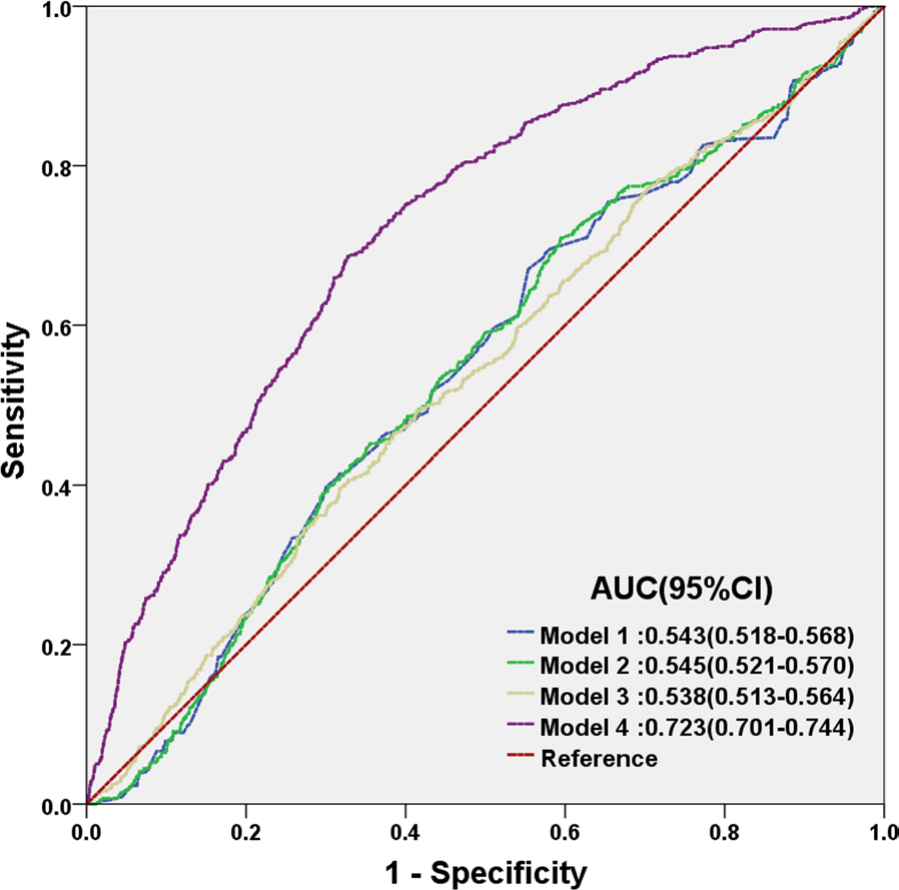
Receiver operator characteristic (ROC) curves for Models 1–4. Model 1: Unadjusted, Model 2: Adjusted for age and gender, Model 3: Adjusted for the same factors as Model 2, plus markers of liver and kidney function. Model 4: Adjusted for the same factors as Model 3, plus metabolic markers. Out of the 4 models, Model 4 has the greatest predictive value, based on its area under the curve (AUC)

The prevalence of NAFLD among patients falling under Q1–5 of UHR increased from 2.4% in Q1 to 17.8% in Q5 (Table 1). Kaplan–Meier analysis showed that increased UHR values was positively correlated with increased risk of NAFLD onset, in which the highest cumulative risk throughout the 5-year follow-up period for NAFLD was found for patients whose UHR fell under Q5 (Fig. 1). This trend suggests that higher UHR are associated with higher likelihood of NAFLD occurrence, which is further supported by multivariate Cox proportional hazard regression analysis, using 4 models. In all 4 models, a significant positive correlation between UHR and NAFLD onset was found, no matter which factors were being adjusted for, or lack thereof, under each of the 4 models for Q2–5, compared to Q1 as the baseline (Table 2). This is particularly evident with model 4, which entails adjustments for age, gender, as well as liver and kidney functional and metabolic indicators. Even with all these adjustments applied, the positive correlation between high UHR and NAFLD onset is still present. Furthermore, model 4 has been shown under ROC curve analyses to be the most predictive for determining NAFLD occurrence, with an area under the curve (AUC) of 0.723 (p < 0.001) (Fig. 2).

**Table 2:** Hazard ratios (HR) for NAFLD onset among the 5 UHR quintile groups for 4 models

Models	Quintile Groups (all HR [95% CI])					*P* value
	Q1	Q2	Q3	Q4	Q5	
Model 1 (unadjusted)	1	1.96 (1.38–2.77)	2.83 (2.04–3.92)	3.72 (2.71–8.83)	6.51 (4.80–8.83)	< 0.001
Model 2 (adjusted for age, gender)	1	1.95 (1.39–2.76)	2.82 (2.03–3.91)	3.69 (2.69–5.07)	6.46 (4.76–9.76)	< 0.001
Model 3 (adjusted as Model 2 + ALP, ALT, AST, ALB, TP, TB, BUN)	1	2.27 (1.44–3.58)	3.70 (2.43–5.66)	4.67 (3.10–7.05)	6.53 (4.38–9.76)	< 0.001
Model 4 (adjusted as Model 3 + FPG, TC, TG, LDL, BMI, SBP)	1	1.33 (0.84–2.11)	1.66 (1.07–2.58)	1.67 (1.08–2.59)	1.76 (1.12–2.75)	< 0.001

### UHR is more predictive of NAFLD onset compared to UA or HDL-C alone

**Fig. 3:**
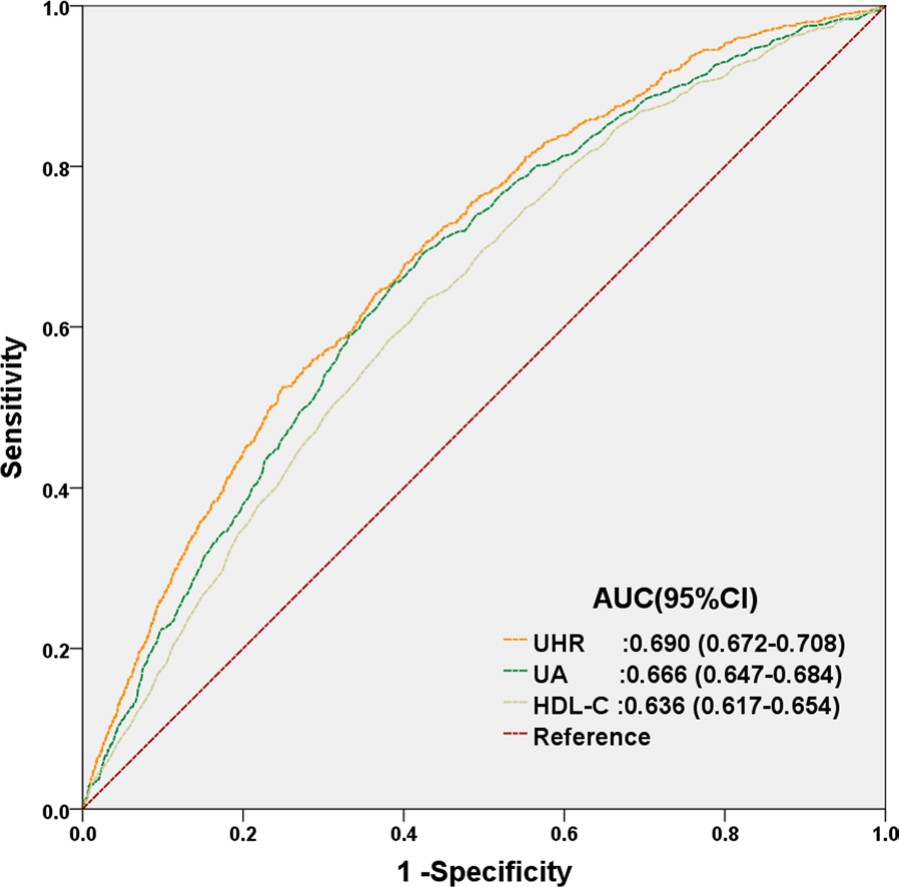
ROC curves for UHR, compared to UA and HDL-C alone. The predictive value for UHR is greater than that for UA or HDL-C alone, as determined by its AUC

ROC curve analyses were then carried out to compare the predictive ability of UHR for NAFLD onset, compared to that of UA and HDL-C alone. The AUC forUHR was higher than forUA or HDL-C, demonstrating that UHR had greater predictive value forNAFLD occurrence, compared to either UA orHDL-C alone (Fig. 3). Furthermore, sensitivity analysis tests were performed on the study participants, excluding those with hyperuricemia (UA > 420 μmol/L in males, > 360 μmol/L in females) and low levels of blood HDL-C (HDL-C < 1.03 mmol/L). Among the 5357 participants found to havenormal UA and HDL-C levels, 518 patients ended up developing NAFLD. Even among these patients, it was found under both univariate and multivariatelogistic regressionanalysis, that higher UHR values was independently associated with increased likelihood of NAFLD occurrence (Table 3). Therefore, UHR not only is better at predicting NAFLDoccurrence than UA or HDL-C levels alone, its predictive value also is applicable even among patients with normal UA and HDL-C levels.

**Table 3:** Univariate and multivariate analyses of factors associated with increased NAFLD onset risk within normal UA and HDL-C ranges

Characteristics	Univariate analysis		Multivariate analysis	
	HR (95% CI)	*P* value	HR (95% CI)	P value
Male Gender	0.416 (0.326–0.530)	** < 0.001**		0.322
Age (years)	1.006 (1.000–1.011)	**0.045**	1.010 (1.003–1.017)	**0.008**
ALP (U/L)	1.015 (1.011–1.019)	** < 0.001**	1.008 (1.003–1.013)	**0.002**
ALT (U/L)	1.025 (1.019–1.031)	** < 0.001**	1.022 (1.009–1.034)	**0.001**
AST (U/L)	1.026 (1.017–1.035)	** < 0.001**		0.111
GLB (g/L)	1.032 (1.008–1.057)	**0.008**		0.506
BUN (mmol/L)	1.074 (1.015–1.137)	**0.014**		0.692
Cr (μmol/L)	1.007 (1.003–1.010)	** < 0.001**		0.201
FPG (mmol/L)	1.689 (1.514–1.884)	** < 0.001**	1.261 (1.110–1.433)	** < 0.001**
TC (mmol/L)	1.596 (1.332–1.913)	** < 0.001**		0.148
TG (mmol/L)	8.960 (6.642–12.09)	** < 0.001**	3.057 (2.047–4.555)	** < 0.001**
LDL-C (mmol/L)	3.106 (2.460–3.923)	** < 0.001**	2.019 (1.229–3.315)	**0.006**
BMI (kg/m2)	1.957 (1.836–2.087)	** < 0.001**	1.641 (1.523–1.768)	** < 0.001**
SBP (mmHg)	1.030 (1.025–1.035)	** < 0.001**		0.162
DBP (mmHg)	1.053 (1.044–1.062)	** < 0.001**	1.023 (1.007–1.038)	**0.004**
UHR (%)	1.008 (1.007–1.009)	** < 0.001**	1.002 (1.000–1.004)	**0.040**

### Comparing the predictive value of UHR with other significant predictors for NAFLD

**Fig. 4:**
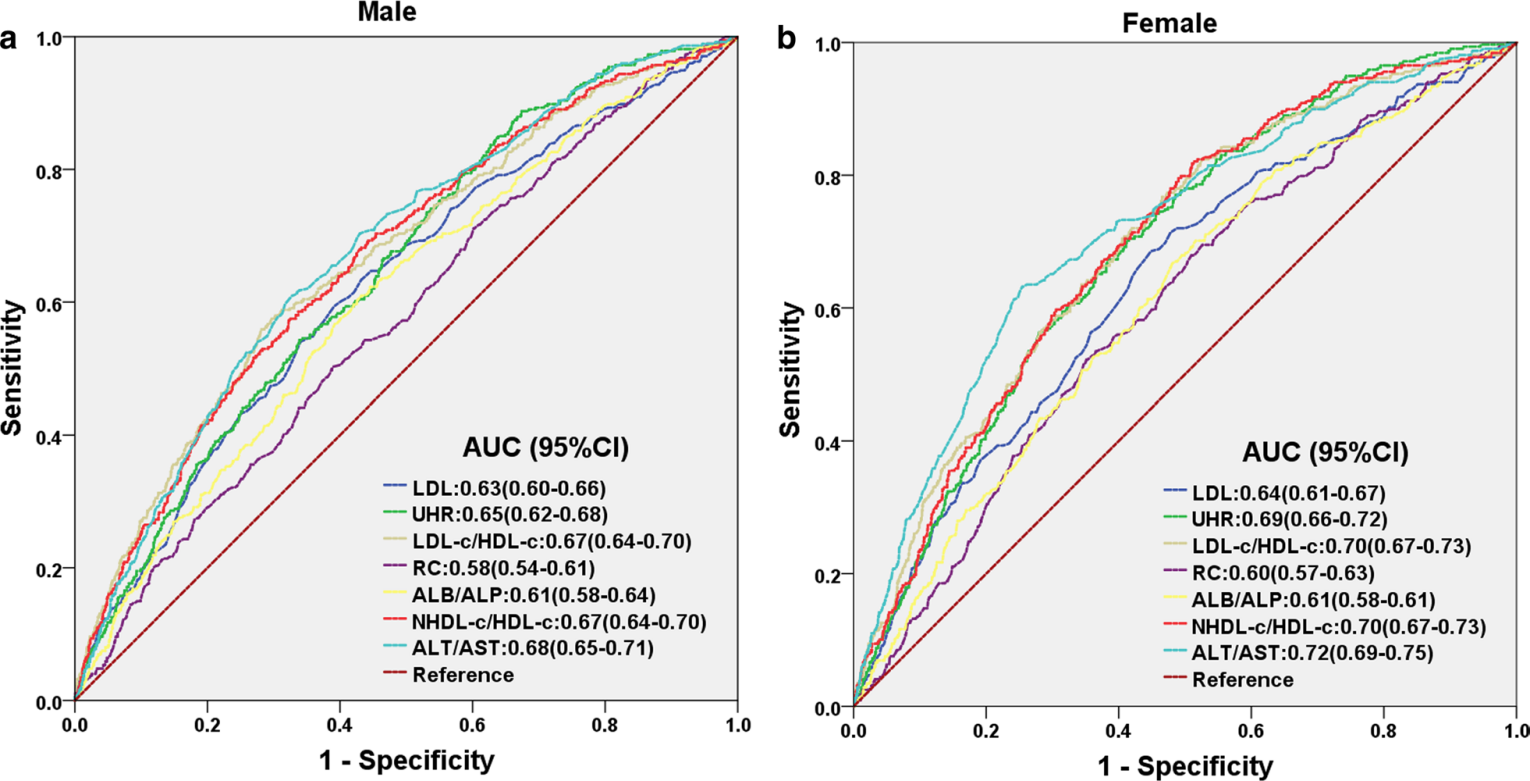
ROC curves for UHR, compared to various other predictive parameters for NAFLD onset, among both males and females. The predictive value for UHR is comparable to, or greater than those other factors, no matter the gender, as determined by its AUC

**Fig. 5:**
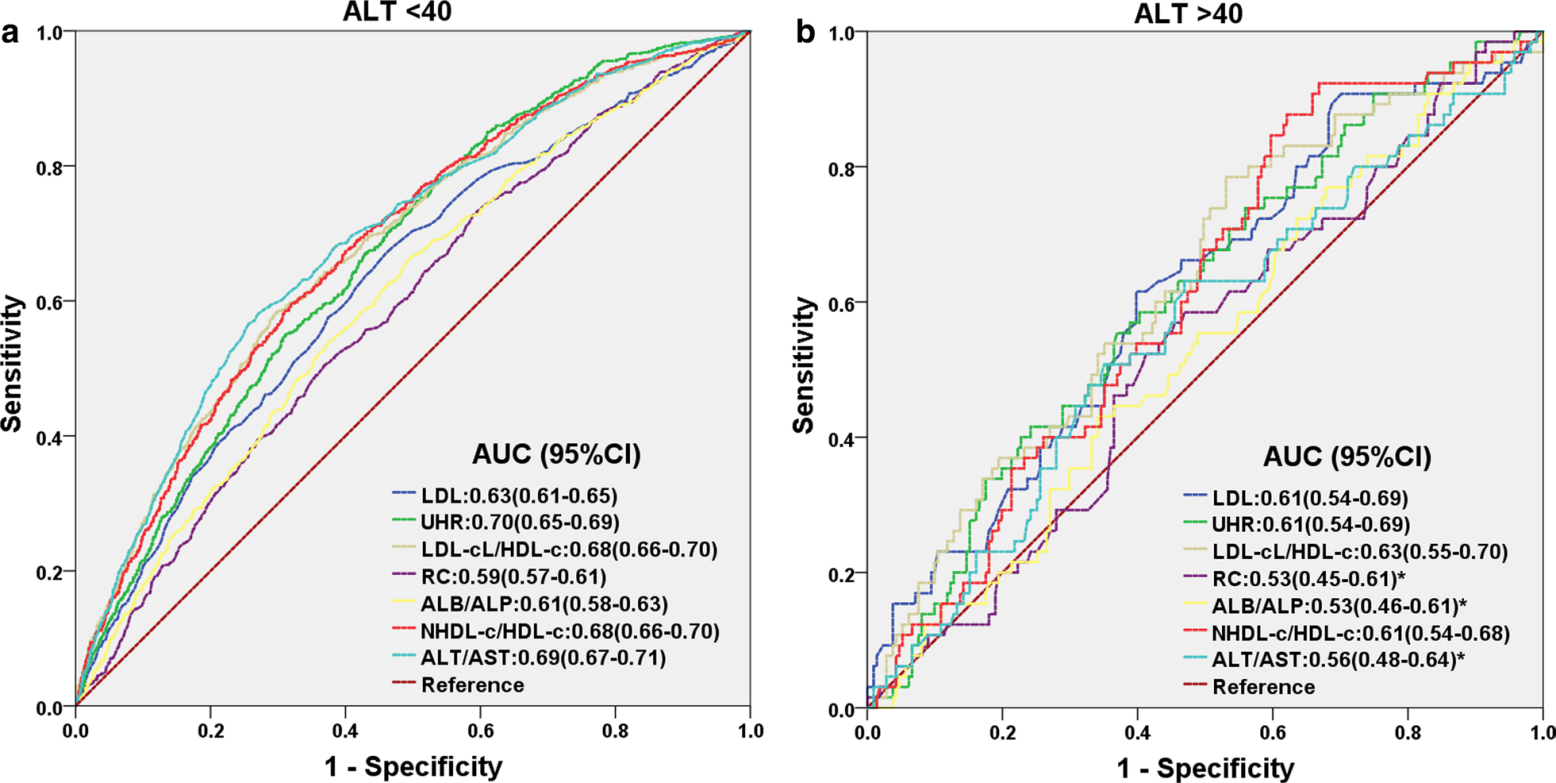
ROC curves for UHR, compared to various other predictive parameters for NAFLD onset, between patients with alanine aminotransferase (ALT), a marker of liver functioning, < 40 U/L, versus > 40 U/L. The predictive value for UHR is retained between the 2 different categories, unlike for remnant cholesterol (RC) (P = 0.441), albumin/alkaline phosphatase ratio (ALB/ALP) (P = 0.419) and ALT/aspartate aminotransferase (AST) ratio (P = 0.159) under ALT > 40 U/L conditions

The predictive value of UHR for NAFLD onset among non-obese Chinese adults was also compared with other significant predictors found in the literature via ROC curve analyses (Table 4). There, it was found that UHR had similar predictive value to LDL-C/HDL-C, NHDL-c/HDL-c, and ALT/AST ratios, and had an AUC greater than that of LDL, RC, and ALB/ALP ratio. Furthermore, the sensitivity of UHR (71%) was the highest among all indicators, with a specificity of 57% and cut-off point of 179.90 (Table 4). It is worth noting, though, that the predictive value for all these aforementioned predictors was higher among females than for males, as shown in Table 5. However, the overall trend regarding UHR versus the other predictors was still the same among both genders (Fig. 4). Similarly, for differing ALT levels, the predictive value of UHR was retained for both ALT < 40 U/Land ALT > 40U/L, which was not the case for RC (P = 0.441), as well as ALB/ALP (P = 0.419) and ALT/AST ratios (P = 0.159) under ALT > 40 U/L conditions (Table 5; Fig. 5). Therefore, UHR could be used as a predictive indicator for NAFLD occurrence, no matter the gender, or the biochemical conditions of the patient.

**Table 4:** Areas under the curve (AUC) with 95% CI, sensitivity, specificity, Youden index and cut-off point for UHR and other significant predictors

Predictor	AUC	95%CI	Pvalue	Sensitivity	Specificity	Youden index	Cut-off point
LDL	0.63	0.61–0.65	< 0.001	0.63	0.59	0.221	2.21
UHR	0.67	0.65–0.69	< 0.001	0.71	0.57	0.278	179.90
LDL-C/HDL-C	0.68	0.66–0.70	< 0.001	0.55	0.71	0.261	1.66
RC	0.59	0.57–0.61	< 0.001	0.54	0.58	0.119	0.73
ALB/ALP	0.61	0.59–0.63	< 0.001	0.68	0.50	0.171	1.49
NHDL-C/HDL-C	0.68	0.66–0.70	< 0.001	0.69	0.56	0.250	1.96
ALT/AST	0.70	0.68–0.72	< 0.001	0.62	0.70	0.324	0.86

**Table 5:** AUC for significant predictors under subgroup analysis for gender and liver functioning

	UHR	LDL	RC	LDL-C/HDL-C	ALB/ALP	NHDL-C/HDL-C	ALT/AST
*Female*							
AUC (95%CI)	0.69 (0.66–0.72)	0.64 (0.61–0.67)	0.60 (0.57–0.63)	0.70 (0.67–0.73)	0.61 (0.58–0.61)	0.70 (0.67–0.73)	0.72 (0.69–0.75)
*P* value	** < 0.001**	** < 0.001**	** < 0.001**	** < 0.001**	** < 0.001**	** < 0.001**	** < 0.001**
*Male*							
AUC (95%CI)	0.65 (0.62–0.68)	0.63 (0.60–0.66)	0.58 (0.54–0.61)	0.67 (0.64–0.70)	0.61 (0.58–0.64)	0.67 (0.64–0.70)	0.68 (0.65–0.71)
*P* value	** < 0.001**	** < 0.001**	** < 0.001**	** < 0.001**	** < 0.001**	** < 0.001**	** < 0.001**
*ALT* < *40 U/L*							
AUC (95%CI)	0.70 (0.65–0.69)	0.63 (0.61–0.65)	0.59 (0.57–0.61)	0.68 (0.66–0.70)	0.61 (0.58–0.63)	0.68 (0.66–0.70)	0.69 (0.67–0.71)
*P* value	** < 0.001**	** < 0.001**	** < 0.001**	** < 0.001**	** < 0.001**	** < 0.001**	** < 0.001**
*ALT* > *40 U/L*							
AUC (95%CI)	0.61 (0.54–0.69)	0.61 (0.54–0.69)	0.53 (0.45–0.61)	0.63 (0.55–0.70)	0.53 (0.46–0.61)	0.61 (0.54–0.68)	0.56 (0.48–0.64)
*P *value	**0.007**	**0.006**	0.441	**0.002**	0.419	**0.007**	0.159

## Discussion

Increasing global incidence of NAFLD has often been associated with obesity and metabolic syndrome [15]. However, NAFLD has recently become more common in non-obese people, especially in China [6]. Studies have shown that non-obese people with NAFLD have altered metabolic states, similar to that of obese people with NAFLD [16, 17], as well as being more likely to develop metabolic syndrome than obese NAFLD [18]. Additionally, a retrospective cohort study showed that approximately 1 in 5 non-obese NAFLD patients also developed carotid atherosclerosis [19]. Another large cross-sectional study in an Indian non-obese population demonstrated NAFLD being significantly associated with coronary artery disease [20]. All of these findings have resulted in the proposal of a new descriptor for this phenomenon, metabolic dysfunction-associated fatty liver disease (MAFLD) [21]. Indeed, Sun et al. [8] found that NAFLD could occur among non-obese Chinese individuals with normal LDL-C levels. Therefore, determining the risk factors for NAFLD among non-obese adults is important in order to develop possible interventional strategies. In the present study, we elucidated a possible predictive factor for NAFLD occurrence in non-obese Chinese adults, UHR. We found that its predictive value was significantly greater than either UA or HDL-C alone, and was comparable to, or better than the other predictive factors mentioned in the literature, such as LDL-C/HDL-C, NHDL-c/HDL-c, ALT/AST, LDL, RC, and ALB/ALP. Additionally, this predictive capability was still present even after adjusting for age, gender, and various biochemical factors. Therefore, UHR could serve as a possible predictor for NAFLD in non-obese populations, even with normal UA and LDL-C levels.

UA is a product of liver purine metabolism, and its presence is closely associated to various metabolic diseases, such as gout, cardiovascular disease, hypertriglyceridemia and diabetes mellitus [22–25]. A relationship between NAFLD and UA levels was first mentioned in 2002 [26]. Since then, some studies have confirmed that UA was an independent risk factor for NAFLD [27, 28], in which UA levels was positively correlated with histological liver damage [29]. In fact, hyperuricemia promotes the onset of metabolic syndrome and NAFLD through various mechanisms, including insulin resistance (IR), oxidative stress, and fructose metabolism disorder [30, 31]. Other studies showed that HDL-Cisalso involved in metabolic syndrome, and that NAFLD patients havelow HDL-C levels [32]. On this basis, Kosekli et al. proposed that UHR might serve as a novel reliable marker for predicting NAFLD onset, in which for every increase of 1 mg/dLin UA levels, there was a 21% increase in the risk for NAFLD occurrence [12]. This finding was in accordance with the results of this study, and we also extended the association of increased UHR and NAFLD occurrence to non-obese Chinese patients with normal lipid levels, in which a significant positive correlation was present. We found from Kaplan–Meier and multivariate Cox proportional hazard regression analysis that individuals with the highest UHR values, falling under Q5, had a much higher risk for the occurrence of NAFLD, compared to those with UHR falling under the lower quintiles Q1–4. This relationship between increased UHR and higher NAFLD onset was independent of any other possible confounding factors. In agreement with our study, Zhang et al. [33] found that the predictive ability of UHR was higher than that of UA and HDL-C alone among thin individuals. Indeed, we determined that the AUC of UHR was higher than for UA and HDL-C (0.690 versus 0.666 and 0.636, respectively). Moreover, within the normal range of UA and HDL-C levels, UHR was still independently associated with NAFLD, greatly increasing its clinical application value.

Recently, Zhao et al. [11] performed step-to-multiple logistic regression analysis on 1000 samples and obtained the NAFLD index (NFI), which includes ALT/AST and TG, as a clinical scoring tool for predicting NAFLD. Accordingly, we compared the predictive value of UHR for NAFLD onset in non-obese adults to other indicators recently proposed in the literature [34–38]. The results showed that UHR was comparable, or better, than those indicators with respect to predictive value, and was significantly more sensitive. We then performed subgroup analysis to determine whether there were differences between genders and levels of liver function, and found that gender had little effect on each predictor. However, abnormal liver function, in the form of ALT > 40 U/L, nullified the predictive value of RC, ALB/ALP, and ALT/AST. By contrast, the predictive value of UHR was still present, no matter the gender or level of liver functioning, making it a more reliable indicator for patients with different clinical profiles.

Currently, liver biopsy is considered as the gold standard for assessing the severity of NAFLD steatosis, but it is not suitable for large-scale screening, due to its invasive nature, as well as the possibility of sampling errors [11]. In this study, NAFLD was diagnosed by ultrasound, though this has the limitation of being unable to determine NAFLD severity. However, ultrasound has been widely used for NAFLD screening, due to its advantages of simple operation, low cost, and high degree of safety [39]. As our study focuses on predicting the onset of NAFLD among non-obese patients, rather than diagnosing its severity, we feel that the ultrasound screening method was suitable for our purposes. Future studies, though, will be required to confirm the predictive capability of UHR for NAFLD onset and severity with the liver biopsy method.

There are several limitations to the current study, one of which is its retrospective nature, leading to the study results being subject to biases not present in prospective studies, such as selection bias and incomplete data collection. In particular, the number of individuals with diabetes, which could be a significant confounding factor with respect to the predictive value of UHR, was not mentioned in the original study. In fact, even after applying the exclusion criteria against patients taking anti-diabetic medications, 80 patients in that study had abnormal fasting plasma glucose levels, raising the possibility of individuals with undiagnosed pre-diabetic or diabetic conditions. Furthermore, the original study did not have any documentation with respect to dietary and alcohol consumption patterns for the patients, which could serve as major confounding factors for obtaining UHR values, as they could affect both UA and HDL-C levels. UA levels could also be affected by the administration of UA-lowering medications, which was not provided by the study documentation. Additionally, the study population composing of Chinese people means that the results may not be fully applicable to other ethnic groups, as they may have differences with respect to certain normal physiological and biochemical ranges. Finally, we did not account for the recently proposed concept of MAFLD, as we did not use its diagnostic criteria in this study [40]. These criteria could have assisted in further clarifying the association between UHR values and NAFLD onset.

## Conclusion

To the best of our knowledge, this large sample retrospective cohort study is the first to demonstrate the association between UHR and NAFLD onset in non-obese Chinese individuals with normal blood lipid levels, in which higher UHR values are independently associated with increased risk for NAFLD occurrence. Indeed, the predictive value of UHR is significantly higher than either UA or HDL-C alone, and was at least comparable to other proposed predictive factors mentioned in the literature. Furthermore, this predictive capability is still applicable, even after adjusting for age, gender, and factors associated with liver and kidney functioning, demonstrating that it could serve as an inexpensive and reliable predictor to detect NAFLD.

## Data Availability

Datasets that support the conclusions of this article are available in the [DRYAD] repository (https://datadryad.org).

## Abbreviations

ALB: Albumin
ALP: Alkaline phosphatase
ALT: Alanine aminotransferase
AST: Aspartate aminotransferase
AUC: Area under the curve
BMI: Body mass index
BUN: Blood urea nitrogen
CI: Confidence intervals
Cr: Creatinine
FPG: Fasting plasma glucose
GLB: Globulin
HDL-C: High-density lipoprotein cholesterol
HR: Hazard ratio
LDL-C: Low-density lipoprotein cholesterol
NAFLD: Nonalcoholic fatty liver disease
NHDL-C: Non-high-density lipoprotein cholesterol
RC: Remnant cholesterol
ROC: Receiver operator characteristic
TB: Total bilirubin
TP: Total protein
UA: Uric acid
UHR: Serum uric acid to HDL-cholesterol ratio
